# Evaluation of Electrocardiographic Ventricular Depolarization and Repolarization Variables in Type 1 Diabetes Mellitus

**DOI:** 10.36660/abc.20180343

**Published:** 2020-02

**Authors:** Mehmet Inanır, Yilmaz Gunes, Isa Sincer, Emrah Erdal

**Affiliations:** 1 Abant Izzet Baysal University Hospital, Bolu - Turkey

**Keywords:** Diabetes Complications, Risk Factors, Prevention and Control, Arrhythmias, Cardiac, Electrocardiography/methods

## Abstract

**Background:**

The risk of cardiovascular events and sudden death increases with type 1 diabetes mellitus (T1DM).

**Objective:**

To evaluate electrocardiographic markers of arrhythmias in T1DM patients.

**Methods:**

Electrocardiographic parameters reflecting ventricular depolarization and repolarization, namely, QT, QTc, QTd, QTdc, Tp-e, JT, and JTc intervals and Tp-e/QT and Tp-e/QTc ratios, of 46 patients diagnosed with T1DM were retrospectively analyzed and compared with 46 healthy age-, sex-, and body mass-matched controls. Correlations between T1DM duration, hemoglobin A1c (HbA1c), and ventricular repolarization variables were analyzed. P values lower than 0.05 were considered statistically significant.

**Results:**

Diabetes duration was 16.6 ± 7.1 years, and HbA1c was 10.81% ± 3.27% in the T1DM group. In comparison with the control group, heart rate, QTc, QTd, QTdc, Tp-e and JTc intervals, Tp-e/QT ratio (p < 0.001), and Tp-e/QTc ratio (p = 0.007) were significantly higher in T1DM patients. T1DM duration and HbA1c levels were significantly correlated with QTc, QTd, QTdc, Tp-e, and JTc intervals and Tp-e/QT and Tp-e/QTc ratios.

**Conclusions:**

In T1DM patients, potential electrocardiographic repolarization predictors were significantly increased in correlation with disease duration and HbA1c levels. These findings may contribute to the understanding of sudden cardiac death in patients with T1DM.

## Introduction

Diabetes is a major health problem that is associated with various comorbidities such as hypertension, cardiovascular diseases, metabolic syndrome, and cardiopulmonary diseases. Over long periods of time, it is also a major underlying risk factor for coronary heart disease, heart failure, peripheral artery disease, atrial fibrillation, chronic renal failure, and stroke. It is also associated with an increased mortality risk.^1-4^

The interval between the beginning of the QRS complex and the end of the T wave in the surface electrocardiogram (ECG) reflects ventricular depolarization and repolarization. Cardiac electrical changes during ventricular repolarization may lead to lethal arrhythmias.^5^ Sudden death risk is also increased in type 1 diabetes mellitus (T1DM) subjects.^6^ Accordingly, prolonged repolarization has been speculated to play a role in sudden death among T1DM patients.^6^

In this study, we aimed to evaluate potential ventricular arrhythmia predictors of surface ECG, namely, QT and corrected QT (QTc) intervals, QT dispersion (QTd), corrected QTd (QTdc), Tp-e, JT and JTc intervals, and Tp-e/QT and Tp-e/QTc ratios, in patients with T1DM.

## Methods

### Study population

ECG records of 46 patients with T1DM, who were followed in the endocrinology and metabolism diseases outpatient clinic of our hospital between January 2017 and May 2018, were retrospectively analyzed and compared with the ECG results of 46 age-, sex-, and body mass-matched controls. T1DM was defined according to the American Diabetes Association criteria.^7^

Patients over the age of 45 were not included due to increased probability of unknown atherosclerosis and comorbidities that may affect ECG. Subjects who had history of coronary artery disease, peripheral artery disease, heart failure, structural heart disease, chronic lung disease, liver or renal failure, thyroid disorders, malignancies, electrolyte imbalances, or any other systemic disease and subjects who were using any drug (e.g. betablockers, calcium channel blockers, antidepressant drugs, etc.) other than insulin were excluded. Subjects who had history of ventricular arrhythmias or atrial fibrillation and subjects who had low QRS voltage, increased QRS duration, left-axis deviation, hypertrophic findings, nonspecific flattening of the T waves, left atrial abnormalities, or ST segment depression on ECG were also excluded due to the probable effects of these ECG changes on the measured ECG parameters.

### Electrocardiography

Twelve-lead ECGs were obtained following a 10-minute rest period, with 10 mm/mV amplitude and 25 mm/s rate with standard lead positions in a supine position, using a commercially available machine (Nihon Kohen Cardiofax ECG-1950 VET). Depending on heart rate, there were four to six beats per lead. ECGs were manually measured, using a magnifying glass (TorQ 150 mm Digital Caliper LCD) by two blinded cardiologists who had no information about the patients. QT intervals were taken from the onset of the QRS complex to the end of the T wave, which was defined as its return to the TP baseline. If U waves were present, the QT interval was measured at the nadir of the curve between the T and U waves. The R-R interval was measured and used to compute the heart rate and to correct QT interval (QTc) with Bazett’s Formula.^8^ QT dispersion (QTd) was determined as the difference between the maximum and minimum QT interval in different leads. The Tp-e interval was defined from the peak of T wave to the end of T wave. Measurements of Tp-e interval were performed from precordial leads. Rate QTc and corrected QT dispersion (QTdc) were calculated using Bazett’s formula (QTc = QT/√RR). JT intervals were measured from the end of the QRS complex (J point) to the end of the T wave (JTend interval). JTc was calculated using Bazett’s formula (JTc = JT/√RR). Tp-e/QT and Tp-e/QTc ratios were also calculated. No patient had fewer than nine measurable leads. Intraobserver and interobserver variations for measurements were less than 5%, and the means of the values defined by the cardiologists were used for analysis.

### Statistical analysis

Analyses were carried out using SPSS 20.0 Statistical Package Program for Windows (SPSS Inc, Chicago, Illinois, USA). Quantitative variables are expressed as mean ± standard deviation (SD), and qualitative variables are expressed as numbers and percentages. The Kolmogorov-Smirnov Test was used to determine if the data were normally distributed. ECG parameters were normally distributed, and disease duration and hemoglobin A1c (HbA1c) levels were not normally distributed. Differences between independent groups were assessed by Student t-test for quantitative variables that were normally distributed and chi-square test for qualitative variables. Spearman correlation analysis was used to examine possible associations between T1DM duration, HbA1c, and ventricular repolarization parameters. P values lower than 0.05 were considered statistically significant.

## Results

Mean diabetes duration was 16.6 ± 7.1 years, and mean HbA1c was 10.81% ± 3.27% in the T1DM group. Mean age, systolic blood pressure (BP), diastolic BP, body mass index (BMI), and frequencies of sex, smoking, and hyperlipidemia were not significantly different between study patients and control group (Table 1).

**Table 1:** General characteristics of the study groups

Baseline characteristics	T1DM (n = 46)	Control group (n = 46)	p value
Age (years)	33.8 ± 8.8	33.8 ± 6.2	1.000
Male/female	28/18	28/18	1.000
Systolic BP (mmHg)	124.4 ± 8.7	121.4 ± 6.7	0.069
Diastolic BP (mmHg)	81.0 ± 4.1	78.4 ± 3.3	0.581
Smoking	7 (15.2%)	9 (19.6%)	0.587
Hyperlipidemia	3 (6.5%)	3 (6.5%)	1.000
BMI	24.2 ± 4.7	24.1 ± 4.8	0.955

BMI: body mass index; BP: blood pressure; T1DM: type 1 diabetes mellitus.

In comparison with the control group, heart rate, QTc, QTd, QTdc, Tp-e and JTc intervals, and Tp-e/QT and Tp-e/QTc ratios were significantly higher in T1DM patients (Table 2).

**Table 2:** Electrocardiographic findings of the study population

	T1DM (n = 46)	Control group (n = 46)	p value
Heart rate (bpm)	84.0 ± 16.9	68.3 ± 11.3	< 0.001
QT ms	352.6 ± 27.4	362.4 ± 22.9	0.068
QTc ms	412.9 ± 36.0	384.2 ± 24.6	< 0.001
QTd ms	29.7 ± 13.8	15.2 ± 6.0	< 0.001
QTdc ms	34.5 ± 15.0	16.1 ± 6.6	< 0.001
Tp-e ms	90.3 ± 8.1	74.8 ± 9.9	< 0.001
JT ms	275.1 ± 23.7	278.8 ± 24.7	0.456
JTc ms	321.6 ± 26.0	295.5 ± 24.7	< 0.001
Tp-e/QT	0.26 ± 0.03	0.21 ± 0.03	< 0.001
Tp-e/QTc	0.22 ± 0.03	0.20 ± 0.03	0.007

Bpm: beats per minute; JT: interval from the end of the QRS complex (J point) to the end of the T wave; JTc: corrected JT interval; ms: millisecond; QT: interval from the beginning of the QRS complex to the end of the T wave; QTc: corrected QT interval; QTd: QT dispersion, the difference between the maximum and minimum QT intervals; QTdc: corrected QT dispersion; Tp-e: T-peak to T-end interval.

T1DM duration and HbA1c levels were significantly correlated with QTc, QTd, QTdc, Tp-e and JTc intervals, and Tp-e/QT and Tp-e/QTc ratios (Table 3).

**Table 3:** Correlations of T1DM disease duration and HbA1c levels with electrocardiographic parameters

	T1DM duration (years)	HbA1c (%)
QTc ms	r = 0.417, p < 0.001	r = 0.414, p < 0.001
QTd ms	r = 0.600, p < 0.001	r = 0.353, p < 0.001
QTdc ms	r = 0.669, p < 0.001	r = 0.608, p < 0.001
Tp-e ms	r = 0.606, p < 0.001	r = 0.602, p < 0.001
JTc	r = 0.443, p < 0.001	r = 0.525, p < 0.001
Tp-e/QT	r = 0.615, p < 0.001	r = 0.608, p < 0.001
Tp-e/QTc	r = 0.357, p < 0.001	r = 0.352, p = 0.001

There were no significant correlations between gender, age, BMI, blood pressure, and the measured ECG parameters.

## Discussion

In this study we have found that, in correlation with disease duration and HbA1c levels, QTc, QTd, QTdc, Tp-e, and JTc intervals and Tp-e/QT and Tp-e/QTc ratios on surface ECG, which may be associated with ventricular arrhythmias and sudden death, were significantly increased in T1DM patients. As far as we know, there is no study in the literature that investigates Tp-e and JT intervals or Tp-e/QT and Tp-e/QTc ratios in T1DM patients.

T1DM patients are at major risk for ventricular arrhythmias and sudden cardiac death.^9^ Presence of reentry circuits, triggered activity, and increased autonomy are among possible mechanism for ventricular arrhythmias. The pathophysiological mechanisms behind arrhythmias have not been fully established in diabetic patients. Structural abnormalities caused by prolonged hyperglycemia and increased fibrosis in the myocardium have been speculated.^10,11^ Myocardial fibrosis, cell loss in the living myocardial tissue and myocardial conduction pathways can create a favorable environment for the formation of micro-reentry circuits. Ventricular arrhythmias may also be triggered by the contribution of impaired electrical balance of the heart and increased sympathetic activity.^12,13^

QT, QTc, and QTd have been shown to predict ventricular arrhythmic events and sudden death in various clinical situations.^14,15^ QT interval is an independent predictor of all-cause and cardiovascular mortality in individuals with type 2 diabetes.^16^ QT interval represents the time from beginning of ventricular depolarization to completion of repolarization. Because QT is typically affected by heart rate, the heart rate-corrected QT interval (QTc) has been proposed as a more appropriate measure of QT.^17^ In many cardiovascular and non-cardiovascular diseases, QTc was shown to be increased.^18^ QTc prolongation has been suggested as an independent marker of ventricular arrhythmias, sudden death, and increased mortality in patients with T1DM as well.^17,19-22^ T1DM patients have been shown to present a positive association of QTc prolongation with age, diabetes duration, and poor metabolic control.^23^ Accordingly, we have also found a positive correlation between QTc and T1DM duration and HbA1c levels.

QTd, which is defined as the difference between the maximum and minimum QT interval on surface 12-lead ECG,^24^ represents ventricular repolarization heterogeneity and is reported as a predictor of ventricular arrhythmias.^24,25^ Increased QTd has also been associated with sudden cardiac death.^26,27^ Tokatli et al. reported that QTd was prolonged in patients with type 2 diabetes mellitus in comparison with controls.^28^ In this paper, we found that QTd was significantly increased in T1DM patients as well, in correlation with disease duration and glycemic control measured by HbA1c. Uysal et al. have found that QTc and QTdc were prolonged in children and adolescents with T1DM.^29^ This prolongation, however, was not associated with disease duration and glycemic control, which may be explained by the relatively young age and short disease duration.

QT interval is composed of depolarization and repolarization components, and it is also affected by QRS period.^30^ However, JT interval is the component of the QT interval that reflects ventricular repolarization alone.^31^ It has been suggested that JT interval may be a more specific repolarization marker than the QT interval.^32^ JT interval may also be affected by heart rate. Therefore, JTc may be more appropriate. Accordingly, Alizade et al.^33^ reported that prolonged JTc was associated with ventricular arrhythmias.

Tp-e interval is also a relatively new ECG parameter showing ventricular repolarization. It has been associated with ventricular arrhythmias and sudden death, even in patients with normal QTc.^34,35^ Tp-e/QT ratio has also recently been used as a new electrocardiographic marker for ventricular repolarization,^36^ and it has been reported to be associated with malignant ventricular arrhythmias.^37^

Most studies assessing ventricular depolarization or repolarization abnormalities in T1DM have used QTc duration and QTdc. However, Tp-e, JT, JTc intervals, and Tp-e/QT and Tp-e/QTc ratios have not been studied. We have found that, in addition to QTc and QTdc, Tp-e and JTc intervals and Tp-e/QT and Tp-e/QTc ratios are increased in T1DM subjects, in association with disease duration and HbA1c levels.

### Limitations

Manual calculation of measurements instead of computer-assisted calculations may be a limitation. Automated measurement systems have been developed for QT measurement, but some problems currently exist with these systems.^38^ Manual identification of T-end is also problematic, cardiologist-dependent, and poorly reproducible. Therefore, automated methods may be preferrable.^39^ Long-term ambulatory ECG monitorization methods might be valuable for documenting the association between the surface ECG parameters studied and arrhythmias. The number of patients in our study was relatively small. A larger patient population would provide more precise results. Association between ECG parameters may be evaluated along with other potential mechanisms for sudden death, such as autonomic dysfunction and fibrosis detected by magnetic resonance imaging. Lack of clinical follow up of the patients regarding arrhythmias and sudden death is another important limitation.

## Conclusions

Previous studies have shown that QTc and QTdc prolongation is important in terms of malignant ventricular arrhythmias in patients with T1DM.^40^ However, Tp-e and JTc intervals and Tp-e/QT and Tp-e/QTc ratios had not previously been measured in patients with T1DM. This study shows that these relatively new repolarization indices and potential electrocardiographic predictors of ventricular arrhythmias are significantly increased in T1DM. Further studies are needed to confirm our results. We hope that clinical significance of this finding for the prediction of malignant arrhythmias will be evaluated in future long-term follow-up and large-scale prospective studies.
